# Pea Rust in Western Siberia: Resistant Varieties and Defense Mechanisms

**DOI:** 10.3390/jof12070514

**Published:** 2026-07-13

**Authors:** Lyudmila Plotnikova, Svetlana Kuzmina, Valeria Knaub, Marina Kukoleva

**Affiliations:** Agrotechnological Faculty, Omsk State Agrarian University Named After P. A. Stolypin, 644008 Omsk, Russia; sp.kuzmina@omgau.org (S.K.); vv.knaub@omgau.org (V.K.);

**Keywords:** *Uromyces pisi*, *Pisum sativum*, pea rust, slow rusting, adult resistance, reactive oxygen species, phenols, genetics

## Abstract

Rust, caused by the fungus *Uromyces pisi*, is the most harmful disease of peas in temperate regions. It is necessary to search for sources of resistance with different defense mechanisms in the pea gene pool. A set of 38 *Pisum sativum* accessions of various origin was studied in Western Siberia in 2021–2024. The aim of the research was to assess the accessions in the field and under controlled conditions using seedlings and adult plants, as well as to study the interaction of *U. pisi* with resistant varieties, and to determine genetic control of rust resistance. All accessions showed partial (incomplete) resistance to rust in the field. A set of 10 resistant varieties was used for studying *U. pisi* interaction with peas using cytological methods. The protective mechanisms of Russian varieties led to the inhibition of 50–90% spores on leaf surfaces before penetration into the stomata, and a part of the small colonies died without hypersensitive reaction in the tissues. Hydrogen peroxide and phenolic compounds with red and green autofluorescence appeared by the stage of sporogenesis. Five varieties showed adult resistance to rust. A hybridological analysis revealed monogenic dominant control of resistance in two varieties, and digenic control in two others. The information obtained expands the understanding of the partners’ interaction in the pathosystem ‘*U. pisi*–*P. sativum*’, and can also be used for breeding pea varieties with different resistance mechanisms.

## 1. Introduction

The most significant place in human diet is taken by 12 cultivated species and cereals (rice, corn, wheat, etc.), provide most of the calories for vital activity [1]. The second most valuable group of the population’s diet is represented by *Fabaceae* spp., which are the most important source of plant proteins. Grain legumes are valuable for human nutrition due to their high protein content with essential amino acids, vitamins, and minerals, while fodder legumes are needed for livestock feeding. Legume cultivation is particularly important for developing and low-income countries, where they serve as the primary source of grain for the population and fodder for animals [2]. Additionally, legumes improve soil structure and contribute to nitrogen accumulation by forming complexes with symbiotic nitrogen-fixing bacteria [3]. The main grain legumes, including soybean, pea, bean, lentil, and alfalfa, occupy 12–15% of the world’s arable land [4,5].

Russia is a major producer and exporter of grain legumes, including peas, soybeans, chickpeas, and lentils. The grain yield of legumes in 2025 was estimated to be 7.5 million tons, including 5.6 million tons of peas. The main legumes importers are China, India, Turkey, Pakistan, and Bangladesh [6]. Large-scale legume crops are located in the Asian part of Russia, particularly in Western Siberia. The Omsk region is located in the south of Western Siberia and specializes in grain production. Grain crops in the Omsk region occupy about 2 million hectares, with about 70% comprising spring common wheat crops, while 8–9% of the area is occupied by legumes, primarily peas (*Pisum sativum* L.) and vetch (*Vicia sativa* L.) [7]. Pea crops are mainly located in the arid steppe and southern forest–steppe, while spring vetch is grown in the more humid and temperate northern forest–steppe and subtaiga zones.

Global legume production is significantly limited by the negative impact of environmental stress conditions and susceptibility to pests and diseases. Peas are affected by a complex of fungal diseases, of which rust causes the greatest damage [8,9]. Pea rust has caused significant crop losses worldwide since the mid-1980s [10,11]. In recent decades, pea rust has become more prevalent in Russian regions, including the Omsk region [12,13,14,15]. Pea rust is caused by two biotrophic rust fungi, viz. *Uromyces viciae-fabae* (Pers.) J. Schrot (syn. *Uromyces fabae* Pers. de Bary) and *Uromyces pisi* (Pers.) Wint. The distribution of these pathogens varies across different agricultural zones, influenced by climate conditions and a set of cultivated crops. *U. viciae-fabae* dominates in tropical and subtropical regions with warm and humid climates, such as India [16,17]. *U. viciae-fabae* has been observed also in peas, lentils, and beans to varying degrees in Canada, Europe, Ethiopia, and Australia [18,19,20]. *U. pisi* causes pea rust in temperate regions and prevails in Europe [9,21,22,23]. Depending on the rust species, the epidemic cycle may be caused by aeciospores or by urediospores. *U. viciae-fabae* is an autoecious rust fungus, which forms aeciospores, urediospores, and teliospores on the same host plant [24]. *U. pisi* affects peas due to urediospores, and the wintering and intermediate development stage takes place on *Euphorbia cyparrissias* [21,25]. In the case of *U. pisi* infection, pea yields can decrease by up to 30% [10].

Previous studies of a broad set of pea accessions have shown that the plants exhibited a susceptible infection type but differed quantitatively in disease severity [23], i.e., partial resistance was manifested [23,26]. The accessions with partial resistance slow down disease development (slow rusting), which leads to an improvement in grain filling and pea grain yield [27]. The International Maize and Wheat Improvement Center (CIMMYT, Mexico) has bred wheat varieties with slow rusting based on adult resistance genes [28], which have shown durable resistance for several decades. On this basis, the breeding of varieties defended by slow rusting and adult resistance is considered promising for durable crop protection [29,30,31]. For breeding pea varieties with multi-level rust protection, the information about resistant mechanisms against *U. pisi* is important.

One of the most common mechanisms of plant defense against biotrophic fungi is rapid cell death after pathogen invasion, named the hypersensitive reaction (HR). HR has been established in pathosystems of ‘*U. vignae*–*Vigna sinensis* Endl. ex Hassk.’, ‘*U. appendiculatus*–*Phaseolus vulgaris* L.’, ‘*U. viciae-fabae*–*P. vulgaris*’, ‘*U. viciae-fabae*–*Lens culinaris Medik*.’, and ‘*Phakopsora pachyrhizi*–*Glycine max* (L.) Merr.’ [32,33,34,35,36]. At the same time, HR was not manifested in the interactions of *U. ciceris-arietini* with chickpea (*Ciceris arietinum* L.) and *Puccinia arachidis* with groundnut (*Arachis hypogaea* L.) [37,38]. In some interactions of rust fungi with cereals and legume species, the development of pathogens was blocked before the penetration of haustoria into plant cells, which was called “pre-haustorial resistance” [22,39]. The resistance of peas to *U. pisi* was mainly associated with the abortion of a significant part of the colonies due to impaired haustoria development without HR [22,23].

The genetics of peas’ and related species’ resistance to rust is under study. The control of pea resistance to *U. pisi* is considered polygenic [40]. The interaction of rust fungi with other legume species may be under by mono-, di-, and polygenic controls [9,16,25]. There is no information on the genetic control of pea rust resistance of Russian-bred varieties to the Western Siberian population of *U. pisi*. The use of resistant varieties in production is the most economically significant and environmentally friendly method for protecting peas against diseases [8,41]. Despite the success in pea breeding, a set of available resistance sources is still limited. Screening of the global *P. sativum* gene pool is necessary to identify promising parental forms with slow rusting mechanisms [21,41]. In this regard, the aim of the research was to evaluate rust resistance of a set of *P. sativum* accessions under a natural infection background in the field and under controlled conditions, as well as to study the defense mechanisms and determine a genetic control of resistance in selected varieties.

## 2. Materials and Methods

### 2.1. Plant Material

The material for research was a set of 38 accessions of *Pisum sativum* L. of various origins from the collection of the N.I. Vavilov All-Russian Institute of Plant Genetic Resources (Saint Petersburg, Russia) and Russian breeding centers. A set accessions included 7 varieties from North America (USA and Canada), 6 from Western Europe (Germany, France, and Great Britain), 1 variety from Australia, 8 from Eastern Europe (Bulgaria, Belarus, Moldova, and Ukraine), and 16 from Russia (Table 1). This set was formed by preliminary selection based on the characteristics of a shortened growing season and resistance to environmental stresses and diseases. Such varieties are necessary to obtain a stable pea harvest in Western Siberia, which is characterized by a short hot summer season and regular droughts.

### 2.2. Field Trials and Data Assessments

The accessions were phenotyped for rust resistance over four cropping seasons (2021–2024) in Western Siberia (Omsk, Russia; 55.024569 N, 73.310549 E). The seeds were sown in the third ten days of May, in rows 1 m long with 10 plants per raw, and three replications were arranged randomly. Harvesting was carried out in the second ten days of August as the plants matured. The weather conditions during the study period were contrasting, namely 2021 was very arid, 2022 was slightly arid, 2023 was arid, and 2024 was wet (hydrothermal coefficients, HTCs = 0.56, 1.2, 0.89, and 1.59, respectively).

The assessment of pea resistance to rust was carried out in the field under a natural infection background, in dynamics with 8–10-day intervals, and four or five estimations were made. The estimations involved disease severity (DS) and infection type (IT). DS was visually estimated as the percentage of canopy covered by rust [42]. According to the final DS estimates, the samples were classified into resistant/susceptible groups: highly resistant—1–10%; resistant—11–25%; moderately susceptible—26–50%; susceptible—51–75%; and highly susceptible—76–100% [43]. IT was scored according Stakman’s scale [44] as follows: ‘0’—no symptoms, ‘;’—necrotic flecks, ‘1’—tiny pustules without sporulation, ‘2’—small pustules surrounded by necrotic halos, ‘3’—pustules with chlorotic halos, and ‘4’—well-developed pustules without chlorosis or necrosis. ITs ‘0–2’ were considered resistant, and ‘3–4’ were defined as susceptible.

Weather conditions contributed to the most intense pea damage in 2022 and 2024. Based on the results of dynamic DS assessments in these seasons, disease progress graphs were constructed, and the indicator ‘Area Under the Disease Progress Curve’ (AUDPC) was calculated using the formula [45]:

AUDPC = 0.5 · (*x*_1_ + *x*_2_) · (*t*_2_ − *t*_1_) + … 0.5 · (*x*_*n*−1_ + *x*_*n*_) · (*t*_*n*_ − *t*_*n*−1_),

where *x*_1_—DS at first estimation, %; *x*_2_—DS at second estimation, %; *x*_n_—DS at final estimation; (*t*_2_ − *t*_1_)—the number of days between the second and first estimation; (*t_n_* − *t_n_*_−1_)—the number of days between the final and previous estimation; and *n*—the number of estimations.

RI was defined as the ratio of the AUDPC of a particular sample to the AUDPC of the maximum affected in the experiment. The accessions were divided according to RI into groups: 0–10—very highly resistant; 0.11–0.35—highly resistant; 0.36–0.65—moderate resistant; 0.66–0.80—low resistant; and >0.80—susceptible [46].

### 2.3. Experiments Under Controlled Conditions

A set of 10 resistant accessions was used to study the interaction of *U. pisi* with peas, viz. vars. Neistoshchimyi 195, Vityaz, Darunok, Nemchinovskyi 46, Pamjati Hangildina, Samorodok, Fragment, Flagman 8, and Flagman 10, and DSP af tl. The susceptible var. Miko was used as a control. The peas were grown in the growth chamber in pots with a photoperiod of 16 h day/8 h night and light intensity of 160 µmol m^−2^ s^−1^ at plant level. For inoculation of 14-day-old plants, a mixture of three *U. pisi* monopustule isolates, obtained from var. Miko in 2024, was used. The urediospores (2 mg spores per plant) were diluted in pure talc (1:10, *w*/*w*) and were applied to the plants using the inoculation tower.

Plants that were 70 days old were used to study defense mechanisms of adult plants. The plants were inoculated by spraying of the *U. pisi* urediospore suspension (10^4^ spores mL^−1^), which was prepared in tapwater with addition of 0.03% Tween-20 as a wetting agent [23]. A sample of urediospores for infecting adult plants was collected on a susceptible var. Miko in 2024. Inoculated plants (3 replicates, with three plants of each accessions) were kept at 100% relative humidity in darkness for 24 h, and then cultivated at 20 °C [47]. IT and DS were estimated on the adaxial leaf surface 14 days after inoculation (dai). The latent period (LP) was defined as the time (days) from inoculation to the opening of 50% of pustules on the leaves [22].

### 2.4. Cytological Studies of U. pisi Interaction with Resistant Peas

The pea leaves infected with *U. pisi* were used for cytological studies. Leaf pieces were fixed by boiling in a lactofenol mixture (lactic acid:phenol:glycerine:water:96% ethanol = 1:1:1:1:8) after 2, 3, 6, and 12 dai. Differential staining of fungal structures was performed using the modified Shipton and Brown’s method [48]. The leaves were stained with 1% aniline blue (Sigma-Aldrich, Boca Raton, FL, USA) in lactophenol at 60 °C for 0.5 h; then, the coloring was differentiated in a saturated chloral hydrate (Acros, Medley, FL, USA) solution (2.5 g chloral hydrate mL^−1^ H_2_O) for 2–3 h at 60 °C. As a result, the intact fungal structures were stained blue, and the damaged ones were dark blue. The intact plant cells were light blue, and the cells that died as a result of HR were stained dark blue.

The leaf pieces of five infected seedlings or adult plants of each accession were used for cytological studies at each fixation time, and the development of 40–50 spores per variant was studied. The detailed analysis of the early *U. pisi* interaction with pea plants was done after 2 dai, in which the proportion of germinated spores (%), growing tubes with appressoria (%), appressoria on the stomata from their total number (%), and appressoria that formed substomatal vesicles (SV) (%) were calculated. Additionally, the proportion of the colonies that died before sporogenesis, as well as the sizes of dead and developed colonies 12 dai (µm^2^), were determined.

The vital leaf staining with 3,3′-diaminobenzidine tetrachloride (DAB, Sigma-Aldrich, Boca Raton, FL, USA) was used for determining hydrogen peroxide H_2_O_2_ generation in the interactions between *U. pisi* and peas. The aqueous 0.02% DAB was introduced into the leaves using vacuum infiltration and incubated for 30 min. The insoluble cherry formazan was formed in the presence of H_2_O_2_ [49]. After staining, the material was fixed in lactophenol mixture. The accumulation of phenolic substances in the cytoplasm and on the plant cell walls were studied by their autofluorescence in reflected light, with an excitation wave λ_max_ = 355 nm and an emission λ_max_ = 420 nm. In the case of intense cytoplasm destruction, the cells had bright green autofluorescence [50]. Cytological studies were carried out using an ARSTEK E62 light microscope (NINGBO YONGXIN OPTICS Co., Ltd., Hi-Tech Park, Ningbo, China), equipped with a Sony Alpha A6400 APS-C digital camera with a resolution of 24.2 MP/inch (Sony, Tokyo, Japan).

### 2.5. Genetic Analysis of the Resistance of Pea Accessions to Rust

A hybridological analysis of four pea varieties (viz. Nemchinovskyi 46, Pamjati Hangildina, Flagman 8, and Flagman 10) was conducted. The resistant varieties were crossed with the susceptible var. Miko. The F_1_ and F_2_ generation of hybrids were produced in 2022 and 2023, respectively. The development of rust on the parent varieties and F_2_ hybrids was estimated on maturing plants under natural infection background in 2023.

### 2.6. Statistical Data Processing

The mean values and standard errors (*M* ± *SEM*) for all dates were determined. The means in resistance rust indicators were separated by Fisher’s least significant difference (*LSD*) test at a probability level of *p* ≤ 0.05 (*LSD*_0.05_) [51]. An analysis of variance (ANOVA) was used to determine genotype and year variances among phenotypes for the DS, AUDPC, and RI, using R-Studio, R 4.3.3 version software, according to the nonparametric Wilcoxson and Kruskal–Wallis tests. The significance of the calculations was assessed using the *p*-value [51,52]. The χ^2^-test was used to confirm the significance of the hybridological analysis results.

## 3. Results

### 3.1. Field Trials

**Figure 1:**
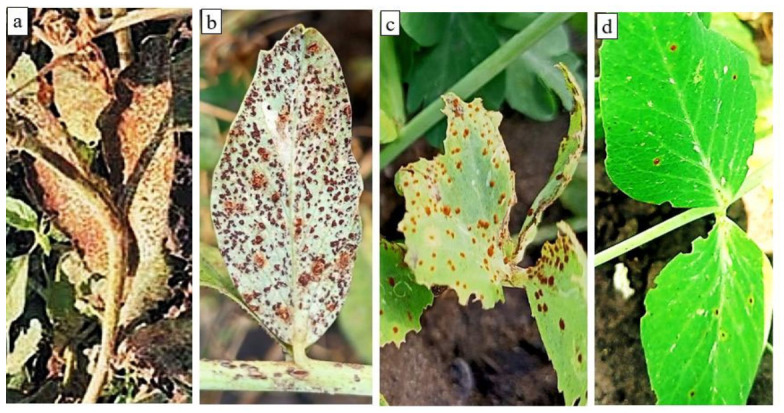
The rust on the pea accessions in Western Siberia (Omsk, Russia): (**a**)—susceptible var. Miko, control, DS 100%; (**b**)—var. Neistoshchimyi 195, DS 80%; (**c**)—var. Flagman 8, DS 20%; (**d**)—var. Flagman 10, DS 1%.

The weather conditions during the research period were contrasting. The growing seasons of 2021–2023 were dry, while in the wet season of 2024, moderate precipitation fell regularly. Weather conditions had a significant impact on the development of rust. In 2021–2023, the first symptoms of the disease appeared on flowering plants during 15–20 July. In 2024, the earliest damage to peas by rust was noted on 25 June on 35-day-old plants, and by the end of July, severe epidemics developed on peas in the region. The orange uredia of *U. pisi* formed first on the abaxial and later on the adaxial sides of the leaves, such as on the stems and pods (Figure 1a–d). By the end of the growing season, telias with black teliospores had formed on the organs of maturing plants (Figure 1b). Aecidia with aeciospores, characteristic for *U. viciae-faba*, were not found on the peas.

**Figure 2:**
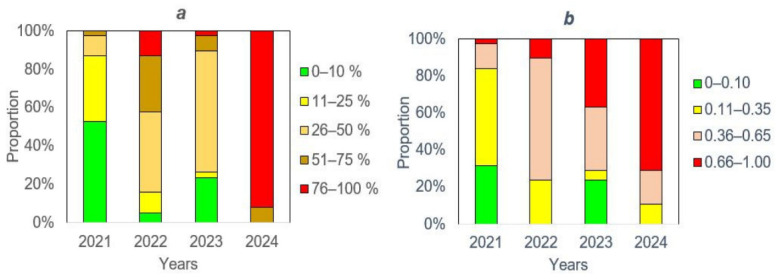
Distribution of pea accessions by disease severity (**a**) and resistance index (**b**) (Omsk, Russia, 2021–2024).

To study the development of *U. pisi* on the accessions, a set of methods was used. Visual observations showed that all the accessions showed susceptible ITs ‘3’–‘4’, and medium-sized urediopustules, surrounded by chlorotic zones or large pustules without chlorosis, had developed on their leaves, i.e., partial resistance was shown (Figure 1a–d). Resistant ITs with HRs (‘0’, ‘1’, ‘2’) were not noted on plants (Table 1). DS assessments were performed as the symptoms of the disease appeared dynamically, and were finished when the samples matured. The var. Miko (control) showed susceptibility in 2021–2024 (final DS = 60–100%), which indicates an intense infection background sufficient to differentiate the samples by resistance. DSs of accessions differed by years, and in accordance with final scoring, the accessions were divided into groups (Table 1, Figure 2a). The ratio of resistant and susceptible accessions changed significantly in 2021–2024. Highly resistant and resistant accessions were identified in 2021 (53 and 34%, respectively) and in 2023 (24 and 3%, respectively), and the average DSs in these years were 14.1% and 34.1%, respectively (Table 1). The increased damage to the accessions in 2022 (average DS = 49.2%) was provoked by small precipitations during pea flowering, and the peas were most affected in 2024 (average DS = 87.9%). In 2024, no resistant varieties were identified by DS, and the proportion of highly susceptible ones reached up to 92%.

In the dry season of 2021, 20 accessions, including American and European varieties, showed high resistance (DS = 0–10%), viz. Venture, Multistar, DSP af tl, Id 29600561, Pois de Clamart nain hatif, Petit Provancol, Azur, and SH 92-793-31-1, as well as 11 varieties of Russian breeding (Table 1). In 2023, only the Russian varieties maintained high resistance, viz. Vitjaz, Darunok, Nemchinovskyi 46, Pamjati Hangildina, Samorodok, Fragment, Flagman 8, Flagman 10, and Orel. Under epidemic conditions of 2024, the DS of most varieties was high (70–100%). The lowest DS (50–60%) was noted for vars. Pamjati Hangildina, Fragment, and Flagman 10.

The disease progress curves were constructed based on the dynamic DS estimates, and the AUDPC and RI indicators were calculated. The average AUDPC increased from 137 (2021) to 1836 (2024) due to suitable conditions for pea damage, such as the increasing number of susceptible accessions (Table 1). The distribution of accessions by DS and RI was similar in 2021, while in 2022–2024, a larger proportion of varieties were classified as highly resistant or resistant by RI (Figure 2b). The DS and AUDPC indicators are commonly used to assess pea resistance to rust in most investigations, and AUDPC is considered the most objective criteria for identifying partially resistant varieties [42,43]. However, DS and AUDPC vary significantly depending on the weather conditions of the growing season; for this reason, the resistance index (RI) was used for facilitating results comparison.

An analysis of variance (ANOVA) was conducted over the period of 2021–2024 to compare the effectiveness of DS, AUDPC, and RI indicators in identifying resistant genotypes in field trials. The resistance scores (DS, AUDPC, and RI) were taken as variable, and the years of field testing (*n* = 4) and genotypes (*n* = 38) were taken as factors (Table 2). ANOVA showed that the yearly conditions was the predominant source of variation for the DS and AUDPC indicators, explaining 72.9% of total variance in field trials, while genotype explained 15.2–19.8% of total variance (*p* < 0.001). However, for the RI indicator, the genotype determines 41.6%, while the year factor explains 38.2% of the total variance (*p* < 0.001) (Table 2). Thus, the RI indicator is more effective for identifying pea genotypes with partial resistance to rust compared to DS and AUDPC.

In 2021, the distribution of accessions into groups according to DS and RI almost coincided (Figure 2a,b), and 10 foreign and 10 Russian varieties were included in the group of highly resistant varieties (Table 1). By 2023, most foreign varieties had become moderately resistant (RI = 0.36–0.65) or had low resistance—susceptible (RI > 0.65), with the exception of a few highly resistant varieties, such as Toledo (Canada) and DSP af tl (USA). Under epidemic conditions in 2024, most North American, Western European, and Eastern European varieties showed low resistance or susceptibility, with the exceptions of Multistar Toledo (Canada), as well as DSP af tl (USA). Six of the Russian varieties (A-Agrimut 767/7, Premium, Berkut, Kaira, Sovinter, and Egorka) lost their resistance by 2023, followed by var. Orel in 2024. The var. Neistoshchimyi 195 maintained moderate resistance (RI = 0.50–0.63) in 2021–2024, and the resistance of the var. Flagman 8 varied from very high to moderate (RI = 0.03–0.62). Vars. Vitjaz, Nemchinovskyi 46, Fragment, and Flagman 10 maintained the most stable resistance over four crop seasons (RI = 0.01–0.33).

**Figure 3:**
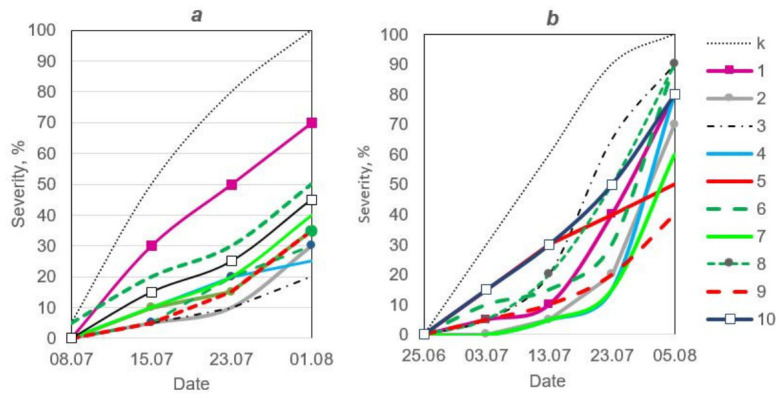
Disease progress curves of pea rust on *P. sativum* accessions: (**a**)—2022; (**b**)—2024. Accessions: k—Miko; 1—Neistoshchimyi 195; 2—Vitjaz; 3—Darunok; 4—Nemchinovskyi 46; 5—Pamjati Hangildina; 6—Samorodok; 7—Fragment; 8—Flagman 8; 9—Flagman 10; 10—DSP af tl.

The dynamics of rust development on 10 varieties that stood out for their resistance (DSP af tl and nine Russian varieties) in 2022 and 2024 are shown in Figure 3. The disease developed at the highest rate on the susceptible var. Miko (Figure 3a,b). All resistant varieties significantly slowed down disease development, and there were quantitative differences in slow rusting between them. In 2022, the slow rusting effect was most pronounced on the vars. Darunok, Vitjaz, Nemchinovskyi 46, Samorodok, and Flagman 10, and to a lesser extent on vars. Flagman 8 and DSP af tl (Figure 3a). Under epidemic conditions in 2024, var. Flagman 10 showed strong slow rusting, while Pamjati Hangildina showed a lesser effect (RI = 0.25 and 0.48). The moderately resistant var. DSP af tl (RI = 0.60) showed weak slow rusting (Figure 3b). The other varieties in the highly resistant group (RI = 0.24–0.29; viz. Fragment, Vitjaz, and Nemchinovskyi 46) showed delayed infection and slow rust progression until July 23, but the disease had intensified by the final assessment. The moderately resistant vars. Neistoshchimyi 195, Samorodok, Darunok, and Flagman 8 were affected earlier, and their DSs increased.

### 3.2. Trials Under Controlled Conditions

The interaction between *U. pisi* and the selected peas was studied using the seedlings and adult plants in a growth chamber. For the experiment, the susceptible var. Miko (control) and 10 varieties with varying degrees of partial resistance (viz. Neistoshchimyi 195, Vitjaz, Darunok, Nemchinovskyi 46, Pamjati Hangildina, Samorodok, Fragment, Flagman 8, Flagman 10, and DSP af tl) were used. The experiments on the seedlings showed that all varieties showed susceptible ITs, with five of them exhibiting large pustules without chlorosis (ITs = ‘4’). For six varieties, *U. pisi* developed both pustules surrounded by a chlorotic zones, and big pustules without chlorosis (ITs = ‘3’, ‘4’) (viz. vars. Nemchinovskyi 46, Pamjati Hangildina, Samorodok, Fragment, Flagman 8, and Flagman 10) (Table 3). The shortest latent period (LP) for rust development was observed for vars. Miko and Nemchinovskyi 46 (LP = 8 days), and LP of the other varieties was 1–3 days longer. The longest LP was noted for the most resistant var. Flagman 10. The susceptible var. Miko was severely affected (DS = 60.8%), while the DS of the other varieties was significantly lower. The lowest DSs were observed for the varieties Vitjaz, Darunok, Fragment, and Flagman 10 (DS = 5.8–9.2%).

Adult resistance to rust was studied using 70-day-old plants. No significant changes in the development of U. pisi were observed on adult plants in comparison with the seedlings of the var. Miko. For most varieties, with the exception of the var. DSP af tl, pustules with ITs ‘3’–‘4’ were detected. LP increased by 1 day, when compared with seedlings, for vars. Neistoshchimyi 195, Vityaz, Darunok, and Nemchinovskyi 46. The DSs of seedlings and adult plants of the var. DSP af tl were similar, while for adult plants of other varieties DSs decreased by 1.4–3.5 times compared to seedlings (Table 3).

### 3.3. Cytological Study of the Interactions Between U. pisi and Resistant Varieties

**Figure 4:**
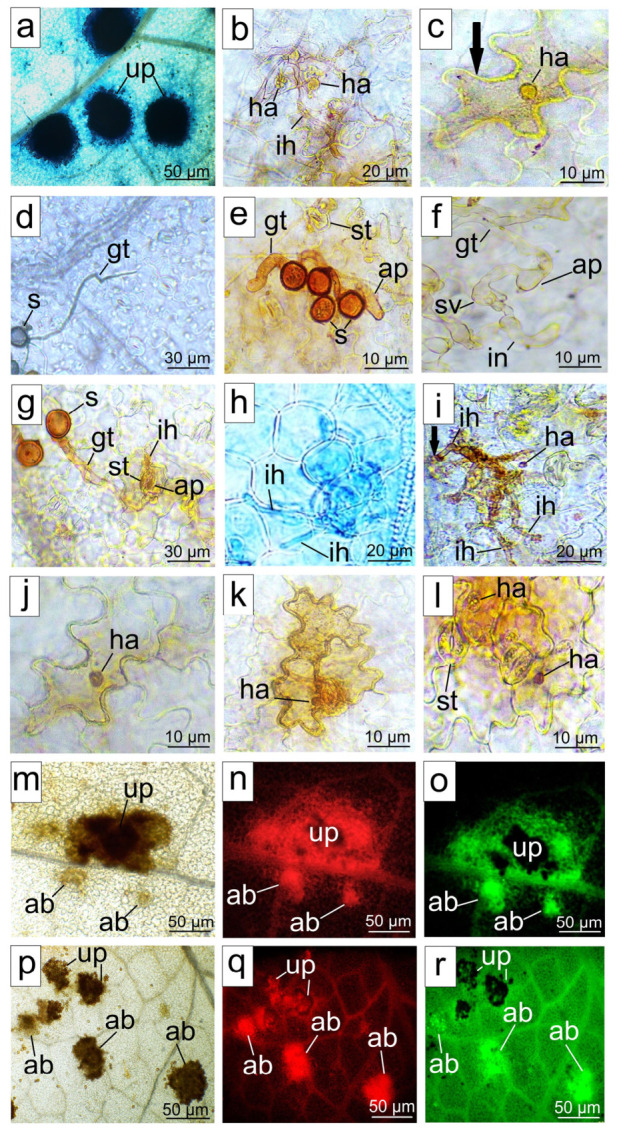
Development of *U. pisi* on the seedlings of pea accessions: (**a**)—urediopustules on the leaf of the var. Miko (control), 12 dai; (**b**)—colony with two haustoria in the tissue of susceptible variety, 2 dai; (**c**)—haustorium in the cell with thickened wall (arrow) in var. Miko; (**d**)—growing tube without appressorium in the leaf of resistant variety, 2 dai; (**e**)—growing tubes and appressorium on the surface of resistant variety, 3 dai; (**f**)—appressorium, substomal vesicle, and infection hypha on the surface of resistant variety, 2 dai; (**g**)—appressorium and the rudimentary infection hypha in resistant variety, 3 dai; (**h**)—small colony with infection hyphae without HMCs in resistant variety, 2 dai; (**i**)—aborted colony with one haustorium and three infection hyphae without HMCs, and with H_2_O_2_ accumulation in the zone of penetration attempt into plant cell (arrow), 6 dai; (**j**)—dead haustorium in the cell, with the traces of H_2_O_2_ but without collapse, 3 dai; (**k**)—H_2_O_2_ accumulation and cell wall thickening in the zone of aborted colony with single haustorium, 3 dai; (**l**)—H_2_O_2_ accumulation in plant cells in the zone of aborted colony in resistant variety, 6 dai; (**m**–**o**)—H_2_O_2_ accumulation and green and red autofluorescence of phenols, corresponding to both urediopustule and abortive colonies in a susceptible variety, 12 dai; (**p**–**r**)—H_2_O_2_ accumulation and phenol green and red autofluorescence, corresponding to both urediopustules and abortive colonies in the resistant var. Flagman 10, 12 dai. Staining: (**a**,**d**,**h**)—aniline blue; (**b**,**c**,**f**,**g**,**i**–**m**)—DAB; (**n**–**r**)—phenol autofluorescence. Abbreviations: ab—abortive colony, ap—appressorium, gt—growing tube, ha—haustorium, ih—infection hypha, s—spore, st—stoma, sv—substomal vesicle, up—urediopustule.

The development of *U. pisi* infection structures and the morphology of affected plant cells, as well as hydrogen peroxide H_2_O_2_ generation and phenolic compounds synthesis, were studied. The spore germination on the seedlings of most varieties was similar to the control, with the exception of a significant decrease on vars. Fragment and Flagman 8 (Table 3). For the susceptible var. Miko, the main portion of growing tubes formed appressoria (88%), and the majority of appressoria (86%) was located on the stomata. The pathogen successfully penetrated into the stomata of the var. Miko and formed colonies. Some of the small colonies (~10%) died off in the early stages of development, but the majority formed large pustules (Figure 4a). Actively developing colonies formed infection hyphae with haustorial mother cells (HMCs) and 2–3 haustoria after 2 dai. In most cases, haustoria penetration did not cause rapid defense reactions in the form of H_2_O_2_ accumulation (Figure 4b). However, in some cases, after the haustoria formation, the walls of the affected cell were thickened in both susceptible and resistant varieties (Figure 4c). The development of *U. pisi* on the surface of the var. Neistoshchimyi 195 seedlings did not significantly differ from the control, but with other varieties, differences were noted. A significant portion of growing tubes did not form appressoria on resistant varieties, or appressoria were located out of stomata (Figure 4d,e). Sometimes, appressoria were not able to provide penetration into the stoma, and substomal vesicles (SVs) and infection hyphae were located on the leaf surface (Figure 4 e,f). In some cases, the fungus stopped at the stages of appressorium on the stoma or after penetration and SV formation, sometimes with a single infection hypha (Figure 4g). H_2_O_2_ accumulated in the fungal structures, remaining on the surface and in the plant cells beneath them after 3 dai.

On the seedlings of the remaining resistant varieties, appressoria formation was suppressed by 1.7–3.0 times compared with the control (Table 3). The greatest disturbance in appressoria development was observed on vars. Vitjaz, Darunok, Nemchinovskyi 46, Samorodok, Fragment, and Flagman 10. The significant proportion of appressoria were located on the leaf surface, which indicates a disturbance in the growing tubes’ orientation to the stomata. On these varieties, the fungus often stopped development at the stage of appressorium, and a smaller portion of appressoria provided penetration into the stomata with SV formation. The listed alterations of *U. pisi* interactions with resistant plants led to the death of the pathogen before penetration of haustoria into plant mesophyll cells, i.e., pre-haustorial resistance was manifested.

**Figure 5:**
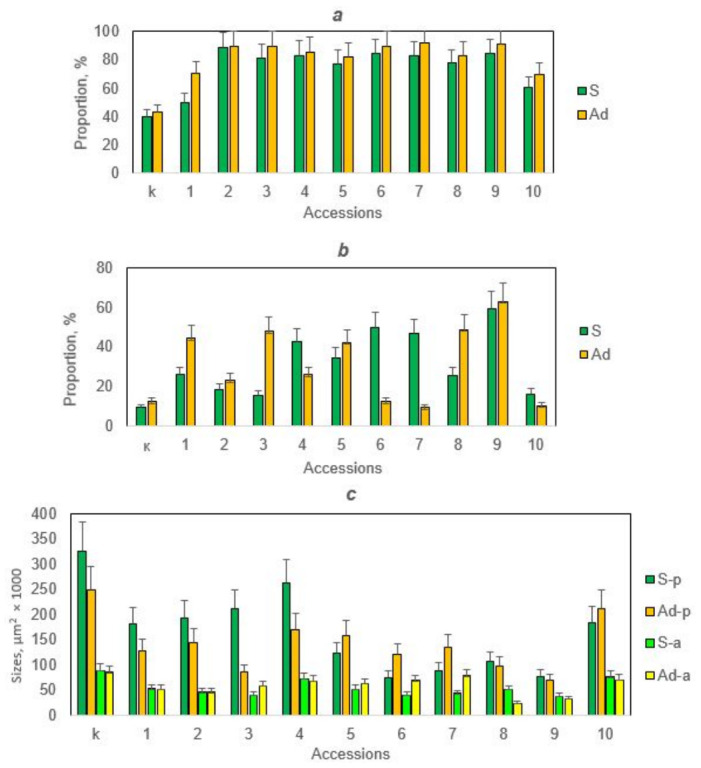
The proportion of *U. pisi* inoculum that died on the surface (**a**), and in the tissues (abortive colonies) (**b**), as well as the sizes of abortive and developed colonies with pustules (**c**) on the seedlings and adult pea plants. Accessions: k—Miko; 1—Neistoshchimyi 195; 2—Vitjaz; 3—Darunok; 4—Nemchinovskyi 46; 5—Pamjati Hangildina; 6—Samorodok; 7—Fragment; 8—Flagman 8; 9—Flagman 10; 10—DSP af tl. Abbrevations: Ad—adult plants; S—seedlings; Ad-a—abortive colonies in adult plants; Ad-p—colonies with pustules in adult plants; S-a—abortive colonies in seedlings; S-p—colonies with pustules in seedlings. The standard error represents the average of five repetitions.

On the adult plants of four varieties (viz. vars. Darunok, Fragment, Flagman 8, and Flagman 10), spore germination was decreased (Table 3). The formation of appressoria did not change significantly compared with the seedlings of vars. Miko, DSP af tl, Vitjaz, Fragment, and Flagman 10. However, for the remaining varieties, appressoria development was moderately suppressed, which was most pronounced with var. Neistoshchimyi 195 (up to 1.4 times). At the same time, for most varieties (excluding vars. Nemchinovskyi 46, Pamjati Hangildina, and Flagman 8), a larger proportion of appressoria was located in the stomata, which indicates an improved orientation of the growing tubes to the stomata in comparison with seedlings. On the most resistant varieties, the proportion of appressoria that formed SVs in the substomal cavities was decreased. As a result of the disruption of fungal structures development on the surface of susceptible var. Miko seedlings, about 40% of the inoculum died (Figure 5a). Inoculum death occurred more intensively on resistant varieties, especially vars. Vityaz, Darunok, Nemchinovskyi 46, Samorodok, Fragment, and Flagship 10 (from 86 up to 90%). On the adult plants of all varieties, inoculum death on the surface increased, which was most pronounced on var. Neistoshchimyi 195 (Figure 5a).

After penetration into the seedling stomata, fungal colonies developed with different intensities. In the tissues of the var. Miko, most colonies formed 2–3 haustoria after 2 dai, while the invaded plant cells showed no signs of damage (Figure 4b). Such colonies progressively developed and formed pustules 8–11 dai. However, in some cases, after the development of a normal-sized haustorium, plant cell walls thickened and traces of H_2_O_2_ were detected in their cytoplasm (Figure 4c). Some colonies died in the early stages of development (abortive colonies) in both susceptible and resistant varieties. In abortive colonies, some of infection hyphae did not form haustorial mother cells (HMCs) or HMCs did not form haustoria (Figure 4h). H_2_O_2_ accumulation was noted in some areas where there were attempts of haustorial penetration into cells (Figure 4i). The inhibition of *U. pisi* development after invasion into 1–3 cells was most pronounced in the tissues of vars. Vitaz, Darunok, Samorodok, Fragment, Flagman 8, and Flagman 10. The colonies with a small number of haustoria died off after 3–5 dai, and H_2_O_2_ accumulated moderately in plant cells contacting them (Figure 4i). In some cases, the haustoria died off after penetration into the cell, while the intense collapse and rapid destruction of the cytoplasm, characteristic for hypersensitive reaction (HR), were not noted (Figure 4j). Such haustoria died off 3–5 dai, and H_2_O_2_ gradually accumulated in the cytoplasm of the affected cells (Figure 4k,l).

Following sporogenesis up to 12 dai, H_2_O_2_ accumulated to a great extent in the zones of developed colonies with pustules, as well as near abortive colonies in seedlings of all varieties (Figure 4m,p). In addition, the substances with red and green autofluorescence were detected in the same zones. Green fluorescence was similar to the glow of lignin in small leaf veins. Bright red and green glows were detected in the cytoplasm of cells damaged after tissue rupture above the pustules (Figure 4n,o). In susceptible plants, fluorescence was manifested mainly near the pustules, and bright red and especially green fluorescence was noted near abortive colonies (Figure 4n,o). In the resistant plants, there was no strong phenol accumulation around large pustules, but intense H_2_O_2_ accumulation and phenols with a bright red and green glows were detected in the zones of small- and medium-sized abortive colonies (Figure 4p–r). Additionally, in all resistant varieties, green autofluorescence was observed throughout the leaves and not just in the colony zones (Figure 4r), which differed from the reactions of susceptible plants. As a result of seedling defense mechanism actions, the differences appeared in the proportion of abortive colonies, as well as the sizes of both abortive and developed colonies in the studied varieties (Figure 5b,c). A high proportion of abortive colonies was found in vars. Nemchinovskyi 46, Pamjati Hangildina, Samorodok, Fragment, and Flagman 8 (45–65%). In all resistant varieties, the sizes of the developed colonies were significantly lower than in the control. The smallest colonies with pustules were formed on the leaves of vars. Pamjati Hangildina, Samorodok, Fragment, Flagman 8, and Flagman 10.

**Figure 6:**
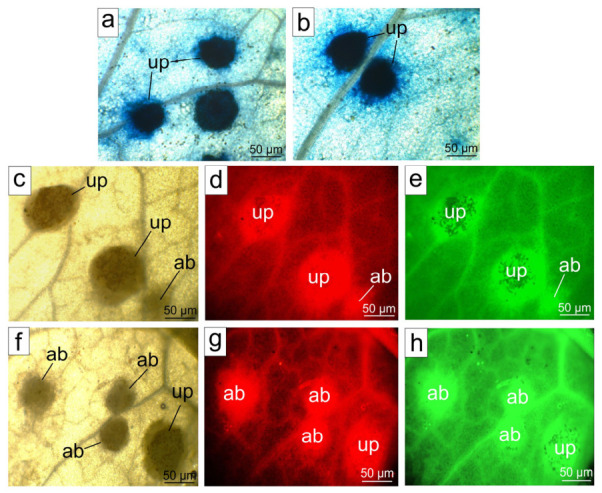
Development of U. pisi in adult pea plants, 12 dai: (**a**)—urediopustules on susceptible var. Miko; (**b**)—urediopustules on adult resistant var. Neistoshchimiy 195; (**c**–**e**)—H_2_O_2_ accumulation and green and red autofluorescence of phenols in the zones of the same urediopustules and abortive colonies in var. Miko; (**f**–**h**)—H_2_O_2_ accumulation and phenols’ green and red autofluorescence in the zones of the same urediopustule and abortive colonies in resistant var. Flagman 10, 12 dai. Staining: (**a**,**b**)—aniline blue; (**c**,**f**)—DAB; (**d**–**h**)—phenol autofluorescence. Abbrevations: ab—abortive colony; up—urediopustule.

In the tissues of the var. Miko’s adult plants, *U. pisi* formed larger colonies and pustules than in resistant varieties (Figure 5c and Figure 6a,b). High H_2_O_2_ accumulation was observed in the zones of all colonies and pustules (Figure 6c,f). The synthesis of phenols with red autofluorescence was more intense in adult plants than in seedlings (Figure 6d,g). The accumulation of phenols with a green glow in the area of pustules and abortive colonies, as well as in leaf tissues as a whole, increased significantly in maturing plants (Figure 6e,h). The green glow was expressed more strongly in resistant plants than in susceptible ones, and especially accumulated in the vars. Darunok, Pamyati Khangildina, Flagman 8, and Flagman 10.

In adult plants of the var. Miko, colonies were 1.4 times less developed than on seedlings, but the proportion of abortive colonies did not change significantly (Figure 5b,c). In adult plants of the var. Neistoshchimyi 195, the sizes of colonies with pustules decreased by 1.7 times, while the proportion of abortive colonies increased by 71%. An even greater decrease in colony sizes (by 2.5 times) and an increase in the proportion of abortive colonies (by 3.1 times) were detected in the var. Darunok. In var. Nemchinovskyi 46, a decrease in colony size (by 1.5 times) was observed, but the proportion of abortive colonies decreased. Small colonies developed on seedlings and adult plants of the var. Flagman 8, and with the age of the plants, the proportion of abortive colonies increased by 1.9 times and their area decreased by 2 times, which indicates an earlier inhibition of the fungus. The most resistant variety, Flagman 10, showed the largest proportion of abortive colonies (more than 60%), as well as the smallest colony sizes with pustules, regardless of the age of the plants. These data indicate that the listed varieties expressed the mechanisms of adult resistance both at the stage of *U. pisi* development on the leaf surface and in the tissues. After penetration into the stomata, the adult resistance was realized, due to which the proportion of abortive colonies increased while the sizes of developed colonies decreased. At the same time, the interactions of *U. pisi* with the adult plants of vars. Samorodok, Fragment, and DSP af tl were different from those described above, as the proportion of abortive colonies decreased sharply and the size of colonies with pustules increased moderately.

Information about the main agronomic traits of the most resistant accessions is important for practical breeding. Russian varieties had different an average “seedlings—full maturity” period in 2021–2024 under contrasting conditions in Western Siberia. The vars. Neistoshimyi 195, Darunok, Pamyati Khangildina, and Nemchinovskii 46 were mid-early (70–73 days), while Flagman 8, Flagman 10, and Nemchinovskii 46 were medium ripened (75–79 days). The vars. Neistoshchimyi 195, Nemchinovskyi 46, and Flagman 10 showed the highest average grain weight per plant (7.1–7.3 g/plant). The vars. Pamjati Hangildina, Flagman 8, and Darunok had lower grain weights (6.5–6.9 g/plant). The early ripened var. Vitjaz (66 days) had a low grain weight (5.4 g/plant). The medium-ripened American var. DSP af tl showed the lowest grain weight (4.5 g/plant).

### 3.4. Genetic Analysis of the Resistance of Pea Accessions to Rust

Information on the genetic control of pea resistance to rust is important for breeding of the varieties. In this regard, a hybridological analysis of four pea varieties with enough stable resistance was carried out. The development of rust on the parent varieties and F_2_ hybrids was estimated on maturing plants under natural infection backgrounds in 2023. The var. Miko showed high susceptibility (DS = 90%) (Table 4), whereas Nemchinovskyi 46, Pamjati Hangildina, Flagman 8, and Flagman 10 were highly resistant (DS = 1–10%). The F_2_ hybrid offspring crossed with vars. Nemchinovskyi 46 and Flagman 10 were divided into two phenotypic classes, viz. highly resistant and susceptible, in a ratio of 3:1, which corresponds to a monogenic dominant inheritance. The F_2_ hybrids of the cross Flagman 8 × Miko were distributed by phenotype in a ratio of 9:3:3:1, which corresponds to a digenic control with independent gene action. In the offspring of the cross Pamyati Hangildina × Miko, a split into two classes was revealed, viz. highly resistant and susceptible, in a ratio of 9:7. This also corresponds to a dihybrid control with possible complementary gene interactions.

## 4. Discussion

Rust diseases affect legumes worldwide and cause significant crop losses [11,42]. In Russia, legume crops have been expanding in recent decades, and protection against rust diseases is becoming very important. In Western Siberia, pea rust has regularly reached epidemic levels in the last decade. The conducted studies showed a tendency of increasing the rust damage of pea accessions caused by *U. pisi* in Western Siberia in 2021–2024. A set of pea accessions of different origins was comprehensively studied as potential sources of resistance. A set of indicators was used to identify accessions with stable resistance, and the advantage of the RI indicator was shown for monitoring resistance and identifying slow rusting varieties. The interaction of U. pisi with the most resistant varieties was studied under controlled conditions, and five varieties with adult resistance were identified. For the first time, the mechanisms of slow rusting and adult resistance in Russian varieties were studied, and the importance of pathogen inhibition on the surface and in tissues, as well as active reactions (generation of H_2_O_2_ and synthesis of phenolic compounds), in their defense was demonstrated. It was shown that the partial resistance of pea varieties to *U. pisi* is controlled by one or two dominant genes.

It is known that pea rust can be caused by two species, *U. pisi* and *U. viciae-fabae*, whose distribution significantly depends on the climatic conditions [11,25]. The climate in the south of Western Siberia is of a sharply continental type, characterized by long, cold winters, during which the soil freezes deeply, and hot, dry summers. In the Omsk region, pea crops are concentrated in the steppe and southern forest–steppe zones, where annual precipitation is less than 220 and 300 mm per year [53]. Only *U. pisi*, which is more adapted to the conditions of moderate latitudes, was detected on pea crops in the Omsk region.

For breeding pea varieties protected against rust, it is necessary to search for new sources of resistance, preferably with different defense mechanisms. Previous studies of the global pea gene pool have not revealed accessions with complete resistance to *U. pisi* (immune); most of these were susceptible to rust, and a small number of varieties showed a slow rusting effect [21,27,40]. Our experiments showed that all accessions had susceptible ITs but were different in terms of DS, i.e., the peas manifested partial resistance, which was assumed previously for the pathosystem *‘U. pisi*–*P. sativum*’ [21,22,23,34,40].

To determine the most promising accessions for further work, a complex array of research methods was used. The AUDPC indicator is considered the most useful for identifying differences in partial resistance to rust and powdery mildew diseases [27,40,54]. The AUDPC value depends on the rate and duration of disease development under different conditions, such as changes in the *U. pisi* population and plant defense mechanisms. The fluctuations in AUDPCs make it difficult to check variety resistance over several growing seasons. The RI indicator and the appropriate scale proposed by the All-Russian Institute of Phytopathology [46] were used for the first time to compare pea rust resistance. The results of ANOVA shows that the RI indicator allows the identification of a greater genotype contribution to phenotypic variability in comparison with DS and AUDPC. This indicates that RI is more objective and convenient for identifying resistant accessions under changing infection backgrounds and weather conditions.

A set of 10 varieties showed predominantly very high or high resistance during 2021–2024 (RI = 0–0.10 or 0.11–0.35). The analysis of the disease progress curves for these samples showed that, under a moderate infection background in 2023, all varieties showed significant slow rusting. Under epidemic conditions in 2024, four varieties retained the slow rusting effect, while for six varieties, the disease appeared belatedly, though DS had increased by the final assessment. It is possible that the more specialized *U. pisi* forms have appeared in the population under conditions favorable for epidemics. Previously, based on long-term monitoring of the *P. triticina* population on common wheat in Russian regions, it was demonstrated that more than half of new virulent pathotypes appearing at the end of growing season were eliminated by the next year, as they had poor fitness to environmental conditions [55]. This leads to the restoration of plant resistance in the following years. In this regard, further monitoring of pea resistance to rust is necessary.

The study of pea rust development under controlled conditions showed that the latent period of the disease was increased in resistant varieties, while DS and colony sizes were reduced. These results are consistent with the previously obtained data on the increase in the latent period and decrease in *U. pisi* pustule density on pea varieties with partial resistance [22]. In general, the studied pea accessions showed the main components of partial resistance, determined by Parlevliet [29] as the basis of durable resistance to diseases.

Information about the emergence of effective mechanisms in resistant varieties is important for breeding of pea varieties with multi-level protection against rust. Cytological studies have shown that slow rusting varieties provide protection through intensive pathogen inhibition on plant surfaces and abortion of significant portions of colonies in tissues, such as active reactions in the form of H_2_O_2_ generation and phenolic compounds synthesis in the late stages of pathogenesis. The germination of *U. pisi* spores was drastically reduced on the leaves of some varieties. However, the more pronounced disorders were alterations of growing tubes’ orientation to the stomata and a strong suppression of appressoria development. A significant part of the appressoria did not provide penetration into the stomata, or infection hyphae developed on the surface but not in the substomal cavity. Overall, these disturbances led to the death of more than 40% of the inoculum on the surface of susceptible seedlings, while with resistant ones, the death of the pathogen was doubled. On adult plants of all resistant varieties, inoculum death due to the above described disorders was more pronounced, but it was partially compensated by a better orientation of growing tubes to the stomata. The most notable mechanisms of adult resistance were increased colony abortion, reduced pustule size, and accumulation of phenolic substances with green autofluorescence. A decrease in adult plant resistance was noted in four varieties, mainly due to a decrease in the proportion of abortive colonies. This can be explained by the fact that spore samples used for infection under controlled conditions included new aggressive clones that appeared in the *U. pisi* population at the end of the 2024 growing season.

Previously, resistance mechanisms that ensure the suppression of rust fungi before penetration into host cells was called ‘pre-haustorial resistance’ [56,57]. The death of non-specialized rust fungi before the intrusion of haustoria into plant cells is characteristic for interactions with non-host species [58]. Based on the interaction of *Puccinia hordei* with resistant species *Hordeum chilense* and barley varieties carrying alien genes, it was shown that the rust fungus was inhibited before penetration into stomata or after the formation of the first infection hypha [57,59]. When studying the interaction of *P. triticina* and *P. graminis* f. sp. *tritici* with non-host species and resistant wheat varieties carrying effective alien genes from *Thinopyrum ponticum* or *Secale cereale*, it was found that pre-haustorial resistance was associated with disorders in the development of fungal structures on plant surfaces, as well as with death at the stages of appressorium or first infection hypha as a result of reactive oxygen species (ROS) generation by stomatal guard cells [50,60,61,62]. In contrast to the interactions of *P. triticina* and *P. graminis* f. sp. *tritici* with introgressed common wheat accessions, there were no signs of destruction of *U. pisi* appressoria after contact with the pea stomata.

Earlier, the examples of disorders of *U. pisi* and *U. viciae-fabae* infection structures on the surface of different legume genotypes prior to penetration into stomata were identified [20,63,64,65,66,67]. However, it was assumed that the alteration of fungal development on the leaf surface is of marginal importance in reducing infection levels within the host species [64]. At the same time, our studies have shown that Russian varieties providing intensive pathogen inhibition on the leaf surface showed very high resistance in seasons with irregular precipitation, as well as under controlled conditions. We can assume that surface defense mechanisms of Russian varieties can serve as a fairly reliable shield against rust in arid regions. At the same time, under humid conditions favorable for the mass reproduction of the pathogen, this protection was largely overcome by repeated plant infection.

Currently, pathogenesis is considered as a dynamic system, the progress of which depends on the signals exchange between partners. Rust fungi need to receive a complex of sequential signals (physical, chemical, and tigmotropic) from host plants for full development [68]. Upon receiving the appropriate signals, the fungal signaling systems are activated, inducing the morphogenesis of appressorium and other infection structures [69]. Recently, the plant cuticle has been considered not only as a physical barrier preventing penetration but also as a source of chemical and physical signals for pathogen interaction with plant [70]. Shortly after the inoculum comes into contact with the plant surface, sticky extracellular material is released, which ensures adhesion to the leaf and stimulates the spore germination [71]. The effect of the chemical composition of cuticular wax on the development of rust fungi was illustrated by the example of *Medicago truncatula* and *Phakopsora pachyrhizi* interactions [71,72]. In *M. truncatula*, a mutation has been identified leading to the loss of function of the gene *Inhibitor of Rust Germ tube differentiation1* (*IRG1*), as well as changes in the composition of epicuticular wax. The *irg1* mutants lack wax crystals on the abaxial leaf surface and have reduced surface hydrophobicity. As a result, spore germination, appressorium differentiation, and penetration into host cells were inhibited on mutant plants [71]. The successful orientation of the growing tubes to the stomata is stimulated by volatile compounds released from the leaf tissues [73].

The resistant varieties used in our experiments were pre-selected for drought resistance and have thick cuticles. It is possible that the chemical composition of cuticles and waxes of resistant varieties differs from that of susceptible ones, which ensures intensive inhibition of *U. pisi* on the plant surface. The suppression of appressoria development was more pronounced on the surface of adult plants, which may be associated with age-related changes in the composition of cuticles and waxes. It is believed that such physical and chemical barriers ensure durable resistance of non-host species to non-specialized pathogens [39]. The defense mechanism identified in Russian varieties, which prevents *U. pisi* from penetrating into the tissues, can be transferred to new resistant pea varieties.

The HR is characteristic for the interaction of many biotrophic fungi with host plants, and develops after the penetration of haustorium into the cell of resistant plant. HR begins with the recognition of pathogen effectors’ elicitor properties by receptors of resistant plants, which stimulates the rapid generation of reactive oxygen species (ROS), including the superoxide anion O_2_^−^ and H_2_O_2_ [74]. The enzyme superoxide dismutase (SOD) converts the superoxide anion into H_2_O_2_, which is an intensive oxidant, as well as a messenger in the NADPH-oxidase signaling system, implemented through a SA-dependent signaling cascade [75,76]. Nitric oxide NO plays the role of a messenger in the NO-synthase signaling system, and is also involved in the implementation of the HR [50]. Signaling systems are involved in organizing a complex of protective reactions, including ROS accumulation, HR realization, synthesis of protective PR-proteins, phenolic substances of various compositions, the strengthening of cell walls, etc. [75,76].

HR is shown in the interactions of *U. appendiculatus* and *U. viciae fabae* with common beans [33,34], as well as in soybeans with *Phakopsora pachyrhizi* [36]. However, HR was not established in the interaction of *U. pisi* with a large set of pea accessions [21,22,23,40]. The most noticeable sign of impaired interaction was an alteration of the haustoria development necessary for fungal nutrition in part of the colonies and their death at the early stages of pathogenesis [21,22,23]. It is known that haustoria act as feeding organs for biotrophic pathogens and actively absorb carbohydrates from plant cells [77]. Using the example of ‘*Vicia faba*–*U. fabae*’ interaction, it was shown an increase in the activity of fungal invertase, which cleaves disaccharide sucrose into glucose and fructose, occurred in the infected tissues [78]. A hexose transporter protein (Hexose Transporter 1, HXT1p), specific for D-glucose and D-fructose, is localized in the haustorial plasma membrane, and provides active hexoses pumping into the haustoria [79]. Pre-haustorial resistance is considered the main protective mechanism, that acts after penetration into stomata and causes the inhibition of the haustoria formation, that leads to abortion of a significant part of colonies without HR. Similar defenses without HRs have been established in the interactions of rust fungi with other legumes, including the pathosystems ‘*U. viciae-fabae*–faba bean’, ‘*U. ciceris-arietini*–chickpea’, and ‘*Puccinia arachidis*–groundnut’ [34,37,80,81].

In our experiments, there was also an alteration of the formation of HMCs and haustoria at the early stages of interaction, which led to colony abortion without HR that was expressed to varying degrees in resistant varieties. Fungus death can be attributed to several reasons, including unstable interaction with *U. pisi* and peas, weak stimulation of HMC development, the prevention of haustoria penetration into the cell, and the defective functioning of the haustoria. Previously, it was shown using the example of *U. vignae* that the development HMCs and haustoria are stimulated by carbohydrate components from the cell wall of susceptible host plants [82]. In some cases, resistant Russian varieties showed H_2_O_2_ accumulation at the sites of attempted cell penetration, indicating the prevention of invasion. The intrusion of haustoria into cells sometimes caused a rapid response in the form of cell wall thickening, which indicates the recognition of the fungus by plants. However, characteristic for HR collapse, intensive H_2_O_2_ accumulation and rapid cytoplasm destruction were not observed. Intensive H_2_O_2_ accumulation was detected only at the late stages of pathogenesis near abortive colonies and under developing pustules. Haustoria in small colonies were often small in size, and their cytoplasm turned dark after a short time, indicating a change in their function. Perhaps such changes are related with defective genes expression in abortive colonies, which are necessary for metabolite transport from host cells to haustoria.

Phenolic substances play an important role in protecting plants from pathogenic fungi. The key enzyme of the phenylpropanoid pathway, phenylalanine ammonia-lyase (PAL) participates in the synthesis of one of the most important compounds—salicylic acid (SA). Phenylpropanoids are involved in strengthening the cell wall due to the synthesis of polymer lignin. Using the example of the interaction of *U. fabae* with *Vicia faba*, it was shown that phenolic substances suppress haustorial formation and cause a decrease in colony size [83]. We have shown for the first time the accumulation of phenols with red and green autofluorescence in pea leaves infected with *U. pisi*. Previously, it was supported by specific staining that such autofluorescense is associated with the phenols synthesis in plant tissues [48].

Using the example of the interaction of leaf rust fungus *P. triticina* with a resistant wheat with the *Lr19* gene, it was shown that the red glow manifested itself in plant cytoplasm soon after an oxidative burst [48], and coincided in time to the accumulation of low molecular weight phenols in tissues [84], including SA [85]. After 2–3 dai, the cytoplasm of cells with high levels of H_2_O_2_ were destroyed as a result of HR, and showed first an orange and then yellow autofluorescence. At the same time, the lignin with a yellow glow was deposited on the cell walls. An intense green glow was characteristic of lignin in the vascular tissues of seedlings [48]. Phenols in the cytoplasm and lignin deposits with green fluorescence were observed around colonies after the use of the inducer of systemic acquired resistance Benzothiadiazole, as well as around abortive colonies in wheat lines with adult resistance genes [86,87]. Lignin, with a green glow, showed a characteristic reaction to syringin residues [48].

In the studied resistant pea varieties, phenols with red and green glows were noted but not with a yellow glow typical for HR results. The green glow was more pronounced near colonies and over the entire leaf area in the resistant seedlings, and was more pronounced in adult plants of vars. Neistoshchimyi 195, Darunok, Nemchinovskyi 46, Flagman 8, and Flagman 10. It is possible that some compounds with a similar spectrum, including phytoalexins, were accumulated in the tissues of resistant peas. Isoflavonoid pisatin (3-hydroxy-7-methoxy-4′,5′-methylenedioxy-chromanocoumarane) is characteristic for *P. sativum* and defends peas from a set of diseases [88,89].

Information about the genetic control of pea resistance to *U. pisi* is important for breeding programs. Due to the fact that pea resistance to *U. pisi* has manifested in a partial form, the polygenic control or incomplete gene expression is assumed [23,27,40]. Polygenic control of rust resistance is established in the interactions of ‘*Arachis hypogaea*–*Puccinia arachidis*’ and ‘*P. fulvum*–*U. pisi*’ [16,38]. At the same time, partial resistance of pea to *U. viciae-fabae* is defined by a single major gene (*Ruf*) [25]. In faba beans, an incomplete resistance of the non-HR type is controlled by two genes, *Uvf-2* and *Uvf-3* [90]. The monogenic control with complete or partial dominance is revealed in other pathosystems, including ‘*P. vulgaris*–*U. appendiculatus*’, ‘*G. max*–*P. pachyrhizi*’, and ‘*P. sativum*–*U. fabae*’ [9]. Previously, monogenic control of partial resistance was also shown in the interactions of *Puccinia* spp. with cereals. The most well-known work on the effect of wheat adult resistance gene to leaf rust *Lr34* was carried out with a single isolate of *P. triticina* named Flamingo [91]. In this work, it was shown that the partial resistance of adult wheat is mainly determined by the death of colonies with a small number of haustoria without HR. Later, the interaction of lines with adult resistance genes *Lr12*, *Lr13*, *Lr22a*, *Lr34*, and *Lr37* that were infected with another set of *P. triticina* isolates was studied. The results showed that all adult resistance genes cause a significant suppression of the appressoria development, the partial death of appressoria as a result of the superoxide anion generation by stomatal guard cells, and colony abortion without HR [87,92]. The partial resistance of studied resistant pea varieties is determined by a similar set of mechanisms, with the exception of an oxidative burst after the contact of appressoria with the stomata. One of the most durably effective wheat genes of adult resistance to stem rust, *Sr2*, provides partial resistance only due to the disruption of haustoria development and abortion of colonies without HR [93]. Similar effects against *P. triticina* manifests with the *Lr23* and *Lr46* genes [94,95]). These examples show that some genes have a pleiotropic effect on pathogenesis and may provide partial resistance to rust diseases.

For the first time, we conducted a hybridological analysis of four pea varieties with the West Siberian population of *U. pisi*, and showed mono- or digenic control of partial resistance to rust. Differences in phenotypic cleavage in the offspring of vars. Pamjati Hangildina and Flagman 8 (9:7 or 9:3:3:1) indicated variations in the gene effects. All-stage resistance of the var. Flagman 10 was determined by single dominant gene, which has a pleiotropic effect and provides intensive partial inhibition of pathogen development on the plant surface and in the tissues, accompanied by the accumulation of phenolic compounds with a green glow (probably phytoalexins).

Three varieties (viz. Nemchinovskyi 46, Flagman 8, and Pamjati Hangildina) exhibited adult resistance, with strong inhibition of fungal structures on the surface of both seedlings and adult plants, as well as increased phenol synthesis in adult plants. The dominant gene of the var. Nemchinovskyi 46 additionally caused a decrease in pustule sizes. Two dominant genes of the var. Flagman 8 significantly increased the death of colonies in tissues and reduced pustule sizes, while two genes in the var. Pamjati Hangildina had a similar but less pronounced effect. The results obtained indicate the different gene actions in these varieties.

The identified varieties with different manifestation of the protective mechanisms can be used as parent forms for breeding peas resistant to rust.

## 5. Conclusions

The article presents the results of a study of a set of 38 pea accessions of different origins in Western Siberia in 2021–2024. The results show a sharp increase in rust damage of peas caused by *U. pisi*, and overcoming the resistance of most accessions in 2022–2024. A set of indicators (IT, DS, AUDPC, and RI) was used to assess the partial resistance of the accessions, and the RI indicator was shown to be advantageous for comparing varieties under changing infection backgrounds. Using RI, a set of 10 resistant varieties (predominantly Russian breeding) was selected in 2021–2024. All varieties exhibited slow rusting in field conditions, while under controlled conditions, five varieties (viz. Neistoshchmyi 195, Vityaz, Nemchinovskyi 46, Pamyati Hangildina, and Flagman 8) demonstrated adult resistance and increased latent periods (LPs), and the var. Flagman 10 was all-stage resistant. Cytological studies revealed that slow rusting and adult resistance were determined by both the intense suppression of *U. pisi* development on the leaf surface and the death of some colonies within the tissues without HR. Partial resistance was accompanied by the accumulation of hydrogen peroxide H_2_O_2_ and phenolic compounds, with different autofluorescence spectra at the sporogenesis stage. The mechanisms of adult resistance manifested themselves in increased colony abortion, reduced pustule size, and increased accumulation of phenols with a green glow. For the first time, it was shown that partial resistance of peas to *U. pisi* is determined by one dominant gene (vars. Flagman 10 and Nemchinovsky 46) or two dominant genes (vars. Pamyati Khangildina and Flagman 8), with different phenotypic effects and differences in the implementation of protective mechanisms at the cellular level.

The obtained information expands the understanding of the interaction in the ‘*U. pisi*–*P. sativum*’ pathosystem. As a result of this work, promising sources of pea rust resistance with different protective mechanisms and genetic control were determined. These results are important for breeding pea varieties with durable rust resistance.

## Figures and Tables

**Table 1:** The rust development on *Pisum sativum* L. accessions in Western Siberia (Omsk, Russia, 2021–2024).

Accession, Origin	VIR Catalog Number	2021			2022			2023			2024		
		Severity, %	AUDPC	RI	Severity, %	AUDPC	RI	Severity, %	AUDPC	RI	Severity, %	AUDPC	RI
Miko—control Canada	k-8755	60	630	1.00	100	1523	1.00	90	1030	1.00	100	2570	1.00
Venture Canada	k-9307	10	94 *	0.15	60	863 *	0.57	40	910 *	0.88	100	2275 *	0.89
Multistar Canada	k-8880	10	88 *	0.14	45	582 *	0.38	40	595 *	0.58	85	1580 *	0.61
Toledo Canada	k-9686	15	173 *	0.27	50	745 *	0.49	35	358 *	0.35	80	1585 *	0.62
Hendersons American Champion USA	k-488	20	108 *	0.17	80	1253 *	0.82	35	420 *	0.41	100	2570	1.00
Sprite USA	k-9706	20	108 *	0.17	45	798 *	0.52	40	689 *	0.67	90	2296 *	0.89
DSP af tl USA	k-9704	10	88 *	0.14	45	528 *	0.35	30	350 *	0.30	80	1538 *	0.60
Ride de Kuightsurce Great Britain	k-509	15	173 *	0.27	70	945 *	0.62	40	465 *	0.45	100	2260 *	0.88
Id 29600561 Australia	k-9552	5	48 *	0.08	40	556 *	0.37	35	563 *	0.55	100	2236 *	0.87
Pois de Clamart nain hatif France	k-395	5	23 *	0.04	60	778 *	0.51	30	361 *	0.38	90	1980 *	0.77
Neve France	k-9346	25	260 *	0.41	80	1130 *	0.74	45	490 *	0.48	100	2193 *	0.85
Petit Provancol France	-	10	92 *	0.15	40	556 *	0.37	35	659 *	0.64	80	1688 *	0.66
Pea Fruhe Provancal ISP France	-	15	215 *	0.34	50	845 *	0.55	40	666 *	0.65	90	2345 *	0.91
Azur Germany	k-9526	10	88 *	0.14	60	698 *	0.46	33	370 *	0.38	100	2113 *	0.82
Boogie Belarus	-	15	173 *	0.27	40	603 *	0.40	30	610 *	0.59	80	1688 *	0.66
Adriana Belarus	-	15	190 *	0.30	60	880 *	0.58	40	885 *	0.86	90	2305 *	0.90
Ewita Belarus	-	15	173 *	0.27	50	845 *	0.55	30	630 *	0.61	80	1688 *	0.66
SH 92-793-31-1 Bulgaria	k-9696	10	98 *	0.16	50	860 *	0.56	45	860 *	0.83	100	2345 *	0.91
Omega Moldova	k-9037	15	215 *	0.34	60	778 *	0.51	25	364 *	0.37	100	2045 *	0.80
Stepovik Ukraine	k-9456	35	352 *	0.56	50	728 *	0.48	60	1010 *	0.98	90	2345 *	0.91
Ovoche divo Ukraine	k-9402	20	115 *	0.18	55	773 *	0.51	50	910 *	0.88	85	2296 *	0.89
Taras 888 Ukraine	k-9376	30	270 *	0.43	60	880 *	0.58	40	910 *	0.88	100	2235 *	0.87
A-Agrimut 767/7 Russia	k-9775	35	338 *	0.54	70	975 *	0.64	60	1030 *	1.00	100	2275 *	0.89
Premium Russia	-	10	88 *	0.14	55	778 *	0.52	50	930 *	0.90	90	1980 *	0.77
Berkut Russia	-	20	118 *	0.19	60	795 *	0.51	50	898 *	0.87	90	2115 *	0.82
Kaira Russia	-	5	48 *	0.08	45	556 *	0.37	50	910 *	0.88	90	2230 *	0.87
Sovinter Russia	-	10	85 *	0.13	50	775 *	0.51	40	888 *	0.86	100	2236 *	0.87
Egorka Russia	-	15	190 *	0.30	55	785 *	0.52	50	898 *	0.87	100	2245 *	0.87
Neistoshchimyi 195 Russia	-	35	348 *	0.55	70	1300 *	0.63	55	610 *	0.59	80	1128 *	0.50
Vitjaz Russia	k-6631	5	48 *	0.08	10	258 *	0.17	5	52 *	0.05	70	735 *	0.32
Darunok Russia	-	5	48 *	0.08	20	213 *	0.14	10	95 *	0.09	90	1580 *	0.69
Nemchinovskyi 46 Russia	k-9518	2	18 *	0.03	25	358 *	0.23	5	43 *	0.04	80	743 *	0.33
Pamjati Hangildina Russia	k-9420	5	40 *	0.06	15	360 *	0.24	10	95 *	0.09	60	1228 *	0.54
Samorodok Russia	-	1	5 *	0.01	30	343 *	0.22	5	70 *	0.07	90	1175 *	0.52
Fragment Russia	-	1	5 *	0.01	10	425 *	0.28	5	25 *	0.02	60	613 *	0.27
Flagman 8 Russia	k-8767	2	18 *	0.03	20	648 *	0.43	5	52 *	0.05	90	1408 *	0.62
Flagman 10 Russia	k-9042	1	13 *	0.02	35	323 *	0.21	1	18 *	0.02	50	638 *	0.28
Orel Russia	k-9039	5	40 *	0.06	30	343 *	0.22	5	70 *	0.07	80	1753 *	0.77
Average	-	14.1	137.2	0.22	49.2	720.8	0.50	34.1	544.3	0.80	87.9	1835.9	0.82
*LSD*_0.05_	-	-	10.6	-	-	85.8	-	-	46.2	-	-	108.3	-

Note: * significant at *p* ≤ 0.05.

**Table 2:** Analysis of variance (ANOVA) of plant genotype and yearly condition effects on the indicators of pea rust resistance.

Indicator	Factor	SS	Df	MS	F-Value	% SS
DS	Genotype	30,040.3	37	811.9	8.12 ***	19.8
	Year	110,767.7	3	10.0	369.3 ***	72.9
	Residuals	11,098.8	111	100.0	-	7.3
AUDPC	Genotype	118,204,24.0	37	319,470.9	4.85 ***	15.2
	Year	60,520,236.0	3	8.3	306.1 ***	75.7
	Residuals	7,315,084.0	111	65,901.7	-	9.1
RI	Genotype	5.7	37	0.15	6.29 ***	41.6
	Year	5.1	3	1.89	70.1 ***	38.2
	Residuals	2.7	111	0.02	-	20.2

Notes: SS—sum of squares; Df—degree of freedom; MS—mean squares. *** Significant difference at *p* < 0.001.

**Table 3:** Indicators of the *U. pisi* development on pea seedlings and adult plants.

Accession	IT, Score	LP, Days	DS, %	Proportion of, %			
				Germinating Spore	Growing Tubes with Aps	Aps on the Stomata from Whole Number	SVs from Aps on the Stomata
Seedlings							
Miko—control	4	8	60.8	85.1	88.2	86.2	95.0
Neistoshchimyi 195	4	9	47.5 *	83.3	89.3	80.4	85.3
Vitjaz	4	9	7.1 *	79.4	33.5 *	66.1 *	66.2 *
Darunok	4	9	9.2 *	82.5	35.2 *	72.3 *	69.4 *
Nemchinovskyi 46	3, 4	8	12.0 *	79.2	36.4 *	79.6	75.1 *
Pamjati Hangildina	3, 4	10	15.5 *	80.4	45.4 *	88.4	71.5 *
Samorodok	3, 4	10	13.2 *	83.0	36.1 *	64.5 *	82.0 *
Fragment	3, 4	10	6.8 *	76.7 *	30.3 *	63.6 *	68.6 *
Flagman 8	3, 4	10	19.4 *	76.2 *	49.2 *	68.1 *	87.1
Flagman 10	3, 4	11	5.8 *	79.0	28.5 *	71.3 *	73.4 *
DSP af tl	4	9	24.0 *	82.3	52.1 *	78.0	86.2
*LSD*_0.05_	-	-	8.2	7.6	9.8	8.6	10.4
Adult plants							
Miko—control	4	8	65.8	86.2	87.3	90.1	93.3
Neistoshchimyi 195	3, 4	10	30.8 *	79.3	61.7 *	84.4	73.4 *
Vitjaz	3, 4	10	5.1 *	80.9	30.1 *	70.2 *	58.6 *
Darunok	3, 4	10	4.5 *	73.9 *	31.5 *	74.5 *	61.5 *
Nemchinovskyi 46	3, 4	9	7.0 *	79.8	30.7 *	81.4 *	73.2 *
Pamjati Hangildina	3, 4	10	6.7 *	77.6	38.3 *	85.6	69.1 *
Samorodok	3, 4	10	6.9 *	79.7	29.6 *	73.0 *	71.3 *
Fragment	3, 4	10	4.8 *	70.1 *	28.5 *	69.1 *	60.6 *
Flagman 8	3, 4	10	5.7 *	68.8 *	41.1 *	72.3 *	85.3
Flagman 10	3, 4	11	2.1 *	70.3 *	25.6 *	72.5 *	70.8 *
DSP af tl	4	9	21.9 *	79.9	54.2 *	83.2	89.1
*LSD*_0.05_	-	-	7.2	9.2	9.8	8.8	9.8

Note: * significant at *p* ≤ 0.05; Ap—appressorium; IT—infection type; LP—latent period; SVs—substomal vesicles.

**Table 4:** Results of the hybridological analysis of pea accessions resistance to rust.

Crosses	Distribution of Plants by Phenotypes in F_2_, Pcs.				Expected Segregation Ratio	χ^2^	
	Highly Resistant DS 0–10%		Susceptible DS > 50%			Experimental	Theoretical
Nemchinovskyi 46 × Miko	38		15		3:1	0.78 *	3.84
Flagman 10 × Miko	42		18		3:1	0.80 *	3.84
Pamyati Hangildina × Miko	35		10		9:7	1.75 *	3.84
Flagman 8 × Miko	30	12	9	4	9:3:3:1	0.56 **	7.82
	Highly resistant DS 0–10%	Resistant DS 11–25%	Moderately susceptible DS 26–50%	Susceptible DS > 50%			

Note: * significant at *p* ≤ 0.05, and ** at *p* ≤ 0.01.

## Data Availability

All the data are available within the article.

## Abbreviations

The following abbreviations are used in this manuscript:

AUDPC	Area under disease progress curve
DAB	3,3′-diaminobenzidine tetrachloride
dai	Days after inoculation
DS	Disease severity
HMC	Haustorial mother cell
HR	Hypersensitive reaction
IT	Infection type
LP	Latent period
RI	Resistance index
ROS	Reactive oxygen species
SA	Salicylic acid
SV	Substomal vesicle
var./vars.	Variety/varieties
